# Dinophyceae can use exudates as weapons against the parasite *Amoebophrya* sp. (Syndiniales)

**DOI:** 10.1038/s43705-021-00035-x

**Published:** 2021-07-12

**Authors:** Marc Long, Dominique Marie, Jeremy Szymczak, Jordan Toullec, Estelle Bigeard, Marc Sourisseau, Mickael Le Gac, Laure Guillou, Cécile Jauzein

**Affiliations:** 1 IFREMER, Centre de Brest, DYNECO Pelagos, F-29280, Plouzané, France; 2 UMR 7144 Sorbonne Université & Centre National pour la Recherche Scientifique, «Adaptation and Diversity in Marine Environment», Team «Ecology of Marine Plankton, ECOMAP», Station Biologique de Roscoff, 29680, Roscoff, France

**Keywords:** Water microbiology, Plant ecology, Microbial ecology

## Abstract

Parasites in the genus *Amoebophrya* sp. infest dinoflagellate hosts in marine ecosystems and can be determining factors in the demise of blooms, including toxic red tides. These parasitic protists, however, rarely cause the total collapse of Dinophyceae blooms. Experimental addition of parasite-resistant Dinophyceae (*Alexandrium minutum* or *Scrippsiella donghaienis*) or exudates into a well-established host-parasite coculture (*Scrippsiella acuminata*-*Amoebophrya* sp.) mitigated parasite success and increased the survival of the sensitive host. This effect was mediated by waterborne molecules without the need for a physical contact. The strength of the parasite defenses varied between dinoflagellate species, and strains of *A. minutum* and was enhanced with increasing resistant host cell concentrations. The addition of resistant strains or exudates never prevented the parasite transmission entirely. Survival time of *Amoebophrya* sp. free-living stages (dinospores) decreased in presence of *A. minutum* but not of *S. donghaienis*. Parasite progeny drastically decreased with both species. Integrity of the dinospore membrane was altered by *A. minutum*, providing a first indication on the mode of action of anti-parasitic molecules. These results demonstrate that extracellular defenses can be an effective strategy against parasites that protects not only the resistant cells producing them, but also the surrounding community.

## Introduction

Parasites, thought to account for half of species richness in some ecosystems, could constitute the unseen majority of species extinctions [1]. The majority of parasites have essential ecological roles by contributing to the balance of ecosystems, limiting invasions and emergence of infectious diseases and contributing to biomass transfer between trophic levels [2–4]. In marine ecosystems, parasites have a predominant role in the planktonic protist interactome, as inferred by sequence-based correlation networks [5], accounting for up to 18% of interactions [6]. Parasites are important contributors to phytoplankton mortality and can sometimes induce the demise of microalgal blooms [7–9].

Amongst marine parasites, the Syndiniales *Amoebophryidae* (also called marine Alveolate group II, or MALVII) is a widely distributed family [10, 11]. This group is ubiquitous in marine waters, including ultra-oligotrophic environments [12] and has been associated with the demise of toxic microalgal species [8, 13–16] in enriched coastal environments. The *Amoebophryidae* life cycle is characterized by a free-swimming stage (dinospores, referred to as zoospores) followed by two, successive, intracellular stages (trophont then sporont) that eventually kill the host and release hundreds of dinospores. Dinospores are flagellated unicellular forms that survive a few hours to a few days in culture [17].

*Amoebophrya* spp. are specialist parasites that require a compatible host to complete their life cycle. The overall consistency in the host spectrum observed within different strains of the same species suggests a genetic determinism underlying host specialization [18]. Many factors can influence the parasitic population dynamic such as physical (e.g., temperature, water column depth, physical mixing) and chemical (e.g., nutrients) parameters [19]. Optimal abiotic conditions for parasitic infection do not always induce the collapse of targeted dinoflagellate blooms, implicating complex biotic interactions as fundamental to the parasite success [19]. Modeling approaches also indicate that parasitic control of dinoflagellate blooms strongly depends upon the plankton community structure (e.g., cell densities, grazing of free-living stages of parasite stages, competition between cells) [20]. Coexistence between resistant and sensitive hosts could affect parasite propagation through different mechanisms, including dilution effects [20, 21] or through cell signaling as suggested in viral infections [9, 22].

Mechanisms of dinoflagellate host resistance against parasites are poorly known. Different strategies have been described to date, including the production of resting stages [23, 24], the production of intracellular anti-parasitic metabolites [25–29], sometimes released into exudates [29]. The release of anti-parasitic compounds (APC) is a strategy that can be classified within the more general term of allelopathy. The term “allelochemical” refers to any secondary metabolite exuded by a microalga that affects the growth of another co-occurring protist [30]. Whether and how the release of APC can influence the dynamics of parasites remains an open question.

This study investigated whether or not co-occurring Dinophyceae, resistant to *Amoebophrya* sp., can affect the dynamics of infection of a sensitive Dinophyceae host. The well-established parasitic couple *Amoebophrya* sp. (A25)—*Scrippsiella* *acuminata* (ST147) [31] was studied in the presence and absence of resistant dinoflagellate host cells or exudates. Two dinoflagellate species, *Scrippsiella donghaienis* and *Alexandrium minutum*, were selected for several reasons: (a) they can form recurrent dense blooms [32–34] and are potential competitors of *S. acuminata*, (b) they co-occur with *S. acuminata* and *Amoebophrya* sp. in the same estuaries [10, 18], (c) they are resistant to *Amoebophrya* sp. (A25) [18] and (d) *A. minutum* cells are producers of allelochemicals with lytic activity against competing protists [35, 36]. The production of allelochemicals by *S. donghaienis* has not been reported. A series of different experimental set-ups were performed to further characterize the interactions. First, we tested the hypothesis that the presence of resistant cells could inhibit the propagation of the infection in cocultures, allowing cell–cell and chemical interactions. To evaluate potential effects of chemical cues upon the interaction, a second set of experiments was performed to study the possible effects of exudates upon the viability of the dinospores and the infection cycle. A third experiment tested the hypothesis that a loss of dinospore viability was linked to *A. minutum* lytic potency.

## Materials and methods

### Biological material

#### Origin of strains and culture conditions

The five hosts and the parasitic strains originated from coastal marine waters of the NE Atlantic Ocean (Table S1). All strains were non-axenic but were cultured under sterile conditions to avoid additional contamination. The parasite *Amoebophrya* sp. strain A25 (RCC4383) was maintained routinely using the sensitive *S. acuminata* clade STR1 (ST147; RCC1627; previously named *S. trochoidea*) as compatible host. Resistant dinoflagellates used in this study were *A. minutum* (strains CCMI1002, Am176 also named RCC749, DA1257) and *S. donghaienis* (strain Sc39 or RCC4714 sampled during an *A. minutum* bloom). Infected and uninfected host cultures were maintained in a medium prepared with seawater (27 of salinity) from the Penzé estuary (France), stored in the dark for several months before being used, filtered to 0.22 µm, autoclaved, and enriched with modified F/2 nutrients (Guillard’s Marine Water Enrichment Solution, Sigma) and 5% (v/v) soil extract [37]. Cultures used for Experiment 3 were prepared using a different medium (K medium [38], seawater from Argenton, France at 35 of salinity) after acclimation of strains. In both cases, a final filtration (0.22 µm pore size filter) under sterile conditions, was done after addition of nutritive solutions. Stock cultures and experiments were incubated under continuous light (90–140 µE m^−2^ s^−1^, light bulb Sylvania Aquastar F18W/174 or EASY LED universal light 438 mm) at 21 ± 1–2 °C. All experiments were performed with plastic flasks (CytoOne vented flasks in polystyrene).

Uninfected hosts were kept in exponential growth phase by diluting 5 volumes of stock culture into 8 volumes of fresh medium every 3–4 days. Infections were propagated by diluting 1:5 (vol:vol) of the infected culture into healthy hosts *S. acuminata* (ST147) every 3–4 days.

#### Synchronization and collection of *Amoebophrya* dinospores

Density and infectivity of dinospores decrease rapidly after release (Table S2); therefore the use of freshly released dinospores helps to maximize infections in the flask. To produce freshly released dinospores, cultures of parasites were synchronized (unless specified) following the protocol [39]. During synchronization, infections were initiated with 3-day-old cultures of *Amoebophrya* from which dinospores were collected after a gentle separation from the remaining host cells (*S. acuminata* ST147) using gravity filtration through nylon filter (5 μm, Whatman). These dinospores were incubated with the exponentially growing host *S. acuminata* (strain ST147) using a 1:2 parasite:host (vol:vol) ratio to encourage infection of host cells. After 24 h of incubation, infected hosts were collected by filtration on a 5 µm nylon filter then resuspended in an equal volume of new medium, to remove remaining free-living dinospores. Three days later, freshly liberated dinospores of the same age (i.e., synchronized) were separated from remaining host cells by filtration as described before. In prior experiments, no effect of dilutions on dinospore survival over 24 h was observed using fresh culture medium, exudates from the healthy host ST147, or exudates from ST147xA25 infected culture (Table S2). Hereafter, filtrates from ST147 cultures in exponential growth were used to adjust densities by dilution.

#### Preparation of microalgal filtrates

Exudates from exponentially growing microalgal strains were collected by filtration (0.2 µm, acetate cellulose membrane, Minisart) using gentle pressure through a syringe. In the present study, dilution of exudates was expressed as equivalent to the microalgal density (corresponding to the theoretical concentration of cells that would have been reached by the initial culture after a similar dilution). Diluted exudates were used immediately for experiments.

### Cell counting methods

#### Flow-cytometry (FCM): cell count and membrane permeability

Densities and individual cell variables (e.g., forward scatter, size scatter, fluorescence signals) were measured using a flow cytometer equipped with 488 nm and 405 nm lasers. A FACSAria flow cytometer (Becton Dickinson) was used in experiments 1 and 2; a Novocyte Advanteon (ACEA Biosciences) was used in experiment 3. Dinophyceae were discriminated from other particles by red chlorophyll autofluorescence. Free-living (dinospores) and late stages of infection of *Amoebophrya* spp. emit a bright green autofluorescence when excited under blue-violet light [23, 40, 41], a proxy of the parasite survival [17]. This natural autofluorescence was used to estimate the density of viable dinospores by FCM using the 405 nm laser.

Intact cell membranes are impermeable to the SytoxGreen (SYTOX Green nucleic acid stain, Invitrogen), but DNA in cells with altered (i.e., permeable) membranes is stained, emitting a bright green fluorescence. Samples were incubated with SytoxGreen (final concentration of 0.05 μM) for 20 min in the dark before measurement.

#### Prevalence of infections (CARD-FISH)

CARD-FISH samples were fixed with paraformaldehyde (1% final concentration) for 1 h (4 °C in the dark) before filtration on a 0.8 µm, polycarbonate filter with a vacuum pump (< 200 mm Hg). Filters were then dehydrated using successive 50%, 80%, and 100% ethanol solutions, dried and stored in the dark at −20 °C. FISH staining was then performed according to [8]. The prevalence was estimated from microscope observations with an Olympus BX-51 epifluorescence microscope (Olympus Optical) equipped with a mercury light source, a Wide Blue filter set (Chroma Technology, VT, USA) and fluorescence filter sets for PI (excitation: 536 nm; emission: 617 nm) and FITC (excitation: 495 nm; emission: 520 nm).

Prevalence was determined by averaging infection counts on a minimum of 80 cells per replicate. Prevalence was characterized in: non-infected host cells, early stage (one or more dinospores of *Amoebophrya* sp. in the cytoplasm), and advanced stages (intermediate and beehive stages) as described in [42]. The progeny count (i.e., the number of dinospores per infected host) was estimated by dividing the maximal concentration of dinospores by the concentration of infected hosts in advanced stages.

### Experimental set-ups

#### Experiment 1: cocultures

**Fig. 1:**
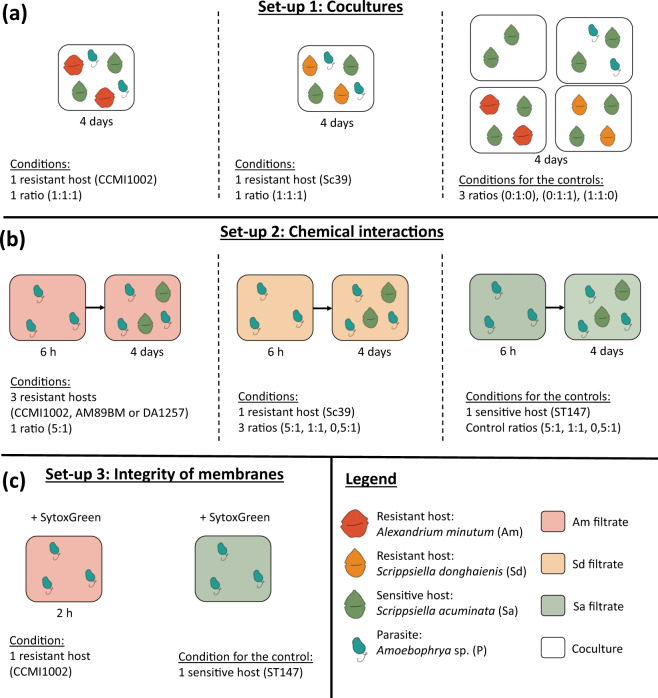
Graphical protocol for the study of chemical defenses against *Amoebophrya* sp. **a** Experimental setup for the coculture experiments. This experiment was conducted over 4 days. The ratios are indicated as (parasite:compatible host:resistant host). **b** Experimental setup for the study of chemical interactions through exudation. This experiment was conducted in two sub-parts, a first pre-exposure to the filtrates over 6 h and an infection of compatible hosts over 4 days. The ratios are indicated as (parasite:compatible host). **c** Experimental protocol for the study of membrane integrity. The exposure to the filtrate was conducted over 2 h and compared to dinospores in their own media.

The dynamic of infection in cocultures was compared when mixing the parasite (*Amoebophrya* sp. A25) with a sensitive host (*S. acuminata* ST147) and a resistant host (*A. minutum* CCMI1002 or *S. donghaienis* Sc39). Mixtures were prepared in triplicates, using a cell ratio *of 1:1:1* (parasite:sensitive host:resistant host), with initial concentrations of 4000 cells mL^−1^ for each strain (Fig. 1a). Controls consisted of flasks containing: (i) only the compatible host ST147 at 4000 cells mL^−1^ or (ii) the host (ST147) at 4000 cells mL^−1^ and parasite A25 at a ratio of 1:1. An additional control consisted of mixing the host ST147 and one of the resistant hosts (CCMI1002 or Sc39) in parallel, replacing the parasite with 0.2 µm filtrate from the host culture. All cultures and controls were started simultaneously, using the same inoculum cultures. Cell densities were quantified once or twice per day. At the end of the experiment, samples were fixed with non-acidic Lugol’s solution (1% final concentration) for microscopic counts and differentiation between *S. accuminata* and *A. minutum* cells.

#### Experiments 2 and 3: evaluation of the effects of Dinophyceae filtrates upon *Amoebophrya*

Filtrates of microalgal cultures were used to analyze the effects of Dinophyceae exudates (from either *A. minutum* or *S. donghaienis*) upon *Amoebophry*a. Experiment 2 was organized into two parts (Fig. 1b): the first to estimate the effect of Dinophycae exudates upon the abundance of autofluorescent dinospores, and the second to analyze the potential for infection and production of a second generation of dinospores after 6 h of contact with the filtrates. The 6 h pre-exposure was chosen to have a stable density of dinospore (Fig. S1) at the moment of the infection.

First, dinospores from *Amoebophrya* sp. (A25) were exposed to dilutions of dinoflagellate filtrates (equivalent to 1,000 and 5,000 and 10,000 cells mL^−1^) collected from three strains of *A. minutum* (DA1257, AM176, CCMI1002) and for one strain of *S. donghaienis* (SC39). Counts of autofluorescent dinospores were monitored by FCM. The mortality rate (h^−1^) of autofluorescent dinospores was calculated over the first 3 h according to Eq. 1, where *N*_1_ and *N*_2_ are the respective densities of autofluorescent dinospores before and after 3 h of exposure to the filtrates. Controls consisted of dinospores incubated with exudates from the host ST147. Incubations for controls and using the highest filtrate concentrations (10,000 cells mL^−1^) were performed in triplicates; whereas only one replicate was performed for intermediate concentrations.

1 $${{{\rm{Mortality}}}}\,{{{\rm{rate}}}} = \frac{{{{{\mathrm{ln}}}}(N_1/N_2)}}{3}$$

Then, dinospores previously exposed to the maximal concentration of exudates and in the control conditions after 6 h of incubation were used for the second part of the experiment (Fig. 1b). Exposed-dinospores were mixed with the host strain ST147 at a theoretical cell ratio of 5:1 (dinospore:host) for dinospores exposed to *A. minutum* filtrates, and at three different ratios (1:2, 1:1, and 5:1) for dinospores exposed to *S. donghaienis* filtrate. These ratios were calculated according to the initial dinospore density before exposure to filtrates and did not consider the possible differential losses related to filtrates. The production of dinospores was monitored twice per day during 5 days by FCM, and prevalence was analyzed after 47 h of incubation by CARD-FISH in the controls and with the CMMI1002 and Sc39 filtrate treatments.

Experiment 3 was performed to monitor the concentrations of autofluorescent dinospores and their membrane integrity over time when mixed with *A. minutum* exudates compared to the control (Fig. 1c). Dinospores from 3-day-old parasite cultures (non-synchronized) of *Amoebophrya* sp. A25 were harvested by filtration (5 µm, cellulose acetate, Minisart). Dinospores were exposed in triplicate to *A. minutum* CCMI1002 filtrate at a final concentration of 5,000 theoretical cells mL^−1^ in six-well plates (CytoOne, polystyrene). In the control, dinospores were diluted in triplicate with *S. acuminata* (ST147) filtrate. The dinospore concentrations and the permeability of their membranes were estimated after 20, 40, 60, and 120 min of incubation with the filtrate.

### Statistics

Statistical analyses were performed using R software [43]. Significant differences in the dependent variables (e.g., concentrations of microalgae and dinospores, prevalence) were assessed with a test of student or one-way ANOVA followed by a post-hoc Tukey HSD, when data met homoscedasticity with a Bartlett test and normality with a Shapiro–Wilk test. When homoscedasticity or normality could not be met, a non-parametric Krukal–Wallis test followed by a post-hoc Conover with a bonferroni adjustment was applied. All tests were performed with a significance level of *p* value = 0.05. Results are expressed as mean ± standard deviation.

## Results

### Infections were mitigated by the presence of a resistant host

**Fig. 2:**
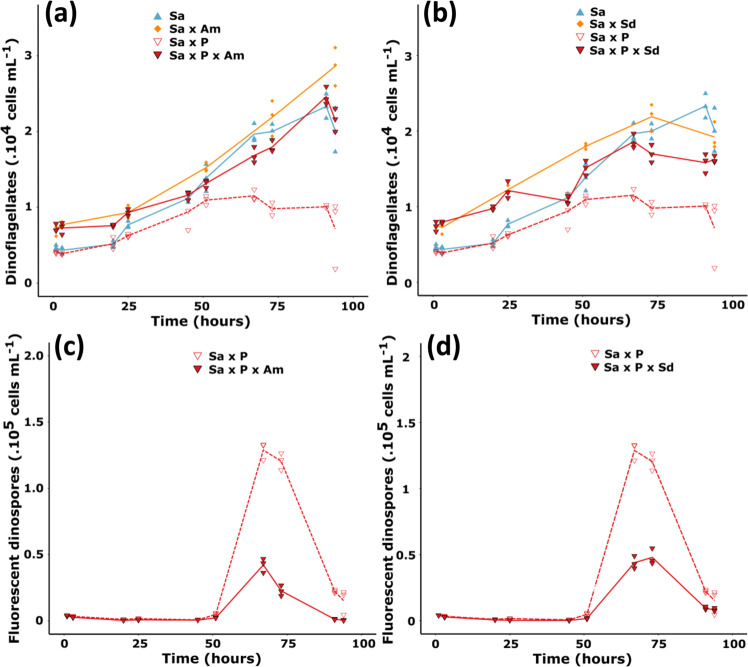
Effect of cocultures on the dynamic of infection. Cocultures of the parasite *Amoebophrya* sp. (P; strain A25) with its compatible host *S. acuminata* (Sa; strain ST147) and a secondary resistant host, either **a**, **b** *A. minutum* (Am; strain CCMI1002) or **c**, **d** *S. donghaienis* (Sd; strain Sc39). Densities of dinoflagellates (*S. accuminata* with *S. donghaienis* or *A. minutum*) are shown in (**a**) and (**c**). Densities of autofluorescent dinospores are shown in (**b**) and (**d**). The same controls (Sa and Sa × P) are shown for both species as experiments were performed at the meantime. Lines represent the mean cell densities while the symbols represent the values of each replicate (*N* = 3).

Experiment 1 tested whether or not the co-presence of a resistant host (*A. minutum* or *S. donghaienis*) could modify *Amoebophrya* infection dynamics with a sensitive host (*S. acuminata*). In controls and when using fixed experimental culture conditions, a complete infection cycle lasted at least 51 h and ended with the sudden released of freshly produced dinospores. During that period, infected host cells do not divide [13], which explain the lower net growth rates recorded 25 h after the parasite inoculation compared to the controls (Fig. 2a, b). Addition of a resistant host (CCMI1002 or Sc39) did not modify the duration of the parasite development, but always resulted in a significant decrease (> 60 %) of dinospore production (Fig. 2c, d). This observation could result from a deleterious effect on the sensitive host, a direct effect upon dinospore survival/infectivity, or both. Cocultivation with *A. minutum* also has a cost for *S. acuminata*. At the end of the experiment, densities of *S. acuminata* in the coculture without parasite were of 6,900 ± 1,400 cells mL^−1^ while it reached 20,000 ± 3,000 cells mL^−1^ in the control.

### Exudates from *A. minutum* decreased the density of viable dinospores

**Fig. 3:**
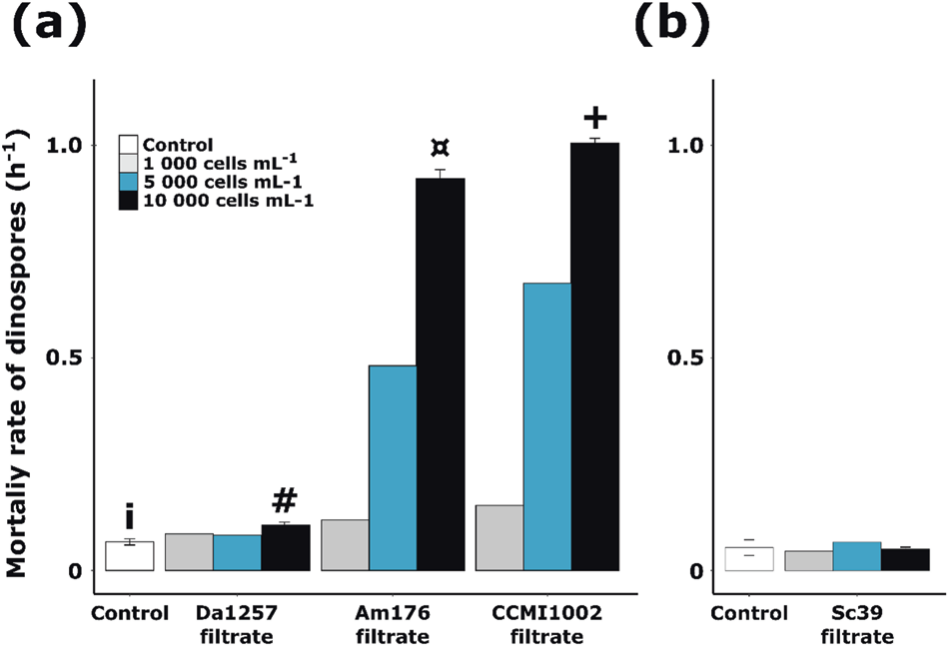
Maximal mortality rate of autofluorescent A25 dinospores in the different conditions. Dinospores were exposed to **a** *A. minutum* and **b** *S. donghaienis* filtrates during two separate sets of experiment. Results are expressed as the value or the mean ± standard deviation when replicates were performed (*N* = 3). Significant differences (*p* value < 0.05) in the mortality rates are indicated by different symbols. The complete dataset, with all sampling points (after 1, 3 and 6 h) is provided in Supporting Information Fig. S1.

Autofluorescence of dinospores can be used as a proxy for their viability [17]. In controls, 25% of fluorescent dinospores were lost after 6 h, leading to a natural mortality rate of 0.07 ± 0.01 h^−1^ in tested cultures conditions (Fig. 3). Experiment 2 tested whether or not resistant dinoflagellate exudates affected mortality rate. If exposure to *A. minutum* filtrates significantly increased mortality (*p* values < 0.02) compared to the control (Fig. 3a), no significant effect using *S. donghaienis* (Sc39) filtrate was observed (Fig. 3b). For *A. minutum*, this deleterious effect was strain-dependent: the mortality rate of dinospores exposed to strain DA1257 (0.11 ± 0.01 h^−1^) was much lower than those measured for AM176 (0.92 ± 0.02 h^−1^) or CCMI1002 (1.00 ± 0.01 h^−1^). This resulted in losses of 32 ± 1%, 96.1 ± 0.2%, and 97.2 ± 0.4%, respectively, of the initial density of autofluorescent dinospores after 6 h of exposure.

### Exudates from *A. minutum* decreased *Amoebophrya* sp. infectivity

To test whether or not the loss of fluorescence (Experiment 2) was linked to a loss of infectivity, dinospores that were challenged for 6 h with exposure to exudates from three strains of *A. minutum* were then mixed with healthy host cultures. Cell densities were fixed for all treatments before the addition of exudates. However, because of the difference in mortality rates, the starting concentration of autofluorescent dinospores and the dinospores:host ratios differed over treatments: 41,000 ± 1,400 dinospores mL^−1^ in the control (ratio 4:1), and 36,000 ± 800,  2,100 ± 100, and 1,500 ± 200 dinospores mL^−1^ with exudates of DA1257 (ratio 4:1), Am176 (ratio 1:5), and CCMI1002 (ratio 1:7), respectively. The ability of the remaining autofluorescent dinospores to infect the host, even at low and unfavorable ratios, then was explored.

**Fig. 4:**
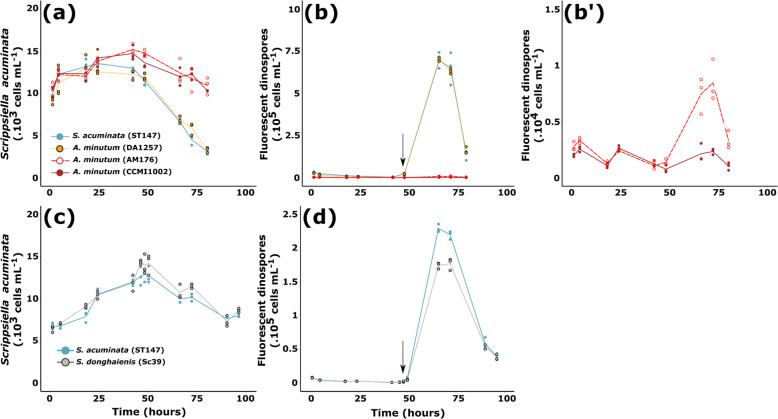
Effect of filtrates on the dynamic of infection. Effect of *A. minutum* (**a**-**b′**) and *S. donghaienis* (**c**, **d**) filtrates (Theoretical cell concentration = 10^4^ cells mL^−1^) on infectivity of *Amoebophrya* sp. dinospores on its sensitive host *S. acuminata* (ST147). Cell densities of *S. acuminata* when mixed with A25 dinospores are shown in (**a**, **d**). Dynamics of dinospores, previously exposed to the different filtrates, when mixed with the compatible host *S. acuminata* ST147 are shown in (**b**, **b′** and **c**. **c** is a zoom of (**b**) with dinospores densities for Am176 and CCMI1002. *S. acuminata* (ST147; blue), *A. minutum* (DA1257; yellow), *A. minutum* (Am176; red) and *A. minutum* (CCMI1002; dark red). In experiments with *S. donghaienis* (Sc39; gray) filtrate, the graphs show results of the experiment at a dinospore: *S. acuminata* ratio of 1:1; results with other ratios can be found in Supporting Information Fig. S2. The arrow represents the sampling point for prevalence analysis which results are shown in Table 1. Lines represent the mean cell densities while the symbols represent the values of each replicate (*N* = 3).

The growth of the compatible host (*S. acuminata* ST147) was suppressed by the dinospores from the control or previously exposed to DA1257 filtrate (Fig. 4a). This suppression of the host growth in the control was linked to the high prevalence (61 ± 6% in the control; Table 1) of *Amoebophrya* sp. in host cells. In comparison, the compatible host in contact with dinospores previously exposed to AM176 or CCMI1002 filtrates remained able to grow during the first 42 h of incubation (Fig. 4a) as the prevalence was lower (approximately a 35% in the CCMI1002 treatment; Table 1). Between 42 and 80 h, a collapse of the host population was observed in all conditions (Fig. 4a). The degree of the decline in host population was likely related to the prevalence of cells at advanced stages of infection (Table 1). With the CCMI1002 treatment, 30 ± 4% of host cell losses were estimated (Fig. 4a) against 75 ± 2% of host cell losses in the control.

**Table 1:** Prevalence of *Amoebophrya* sp. (A25) in *S. acuminata* (ST147) during experiment 2 after 47 h of contact.

	Control	CCMI1002 filtrate	*p* value	Control	Sc39 filtrate	*p* value
*Prevalence (% of host cells)*						
Infected	61 ± 6	35 ± 17	NS	24 ± 16	44 ± 23	NS
Early stages	1 ± 2	16 ± 14	NS	5 ± 9	12 ± 20	NS
Advanced stages	60 ± 5	19 ± 4	***	19 ± 7	33 ± 3	NS (0.07)
Progeny	105 ± 28	1 ± 0	**	43 ± 24	14 ± 3	NS

Two controls are shown as the two experiments were performed during two different sets. Results are expressed as the value or the mean ± standard deviation. Significant values between the control and the dinophyceae treatment (CCMI1002 or Sc39) are indicated as followed: “NS” non significant, “**” 0.01 > *p* value > 0.001, “***”*p* value < 0.001, (*N* = 3).

Novel infections and dinospores releases were observed in all treatments (Fig. 4b). Filtrates of *A. minutum* did not seem to affect the intracellular stage as new progeny were released after 48 h, and the duration of infection was similar over treatments. Progeny (dinospore production per infected host) was 100 times lower with CCMI1002 than in the control (Table 1). As a result of lower prevalence and lower progeny, the maximal dinospore concentration was drastically lower in the CCMI1002 and AM176 treatment (Fig. 4b′) as compared to the control or DA1257 filtrate treatments (*p* values < 10^−7^).

The same experiment was conducted with Sc39, results from the 1:1 ratio are shown in Figs. 4c, d, results from cell ratios of 1:2 and 5:1 are presented in Fig. S2. In contrast to *A. minutum* filtrates, infections started with the same density of autofluorescent dinospores in the controls and in Sc39 treatments, as no effect was observed upon the autofluorescence of dinospores. Filtrates of *S. donghaienis* did not seem to affect the intracellular stage, as novel infections were observed and the duration of infection was similar to control conditions. Release of new progeny started between 48 and 50 h (Fig. 4d). The previous treatment of dinospores with Sc39 filtrate did not significantly affect the prevalence of *Amoebophrya* sp. (Table 1) nor affect the growth rate of the host during the first 48 h (Fig. 4c). With or without the previous treatment with *S. donghaienis* filtrate, a sharp decline in host population, concomitant with release of new progeny, was observed after 48 h. Overall there was no statistical difference in the percentage of lysed host cells between the treatments ST147 (37 ± 3%), and Sc39 (38 ± 4%). The main effect of pre-exposure of dinospores to Sc39 filtrate was observed in the new generation of dinospores: the treatment significantly decreased by 22% the maximum concentration of the new generation of dinospores (Fig. 4d and Supporting Information Fig. S2). This decrease did not seem to be linked to a lower prevalence but was more likely related to a lower number of progeny per infected host, even though the threefold decrease was not statistically significant when compared to the control (Table 1).

### Exudates from *A. minutum* disrupted membranes of *Amoebophrya* sp

**Fig. 5:**
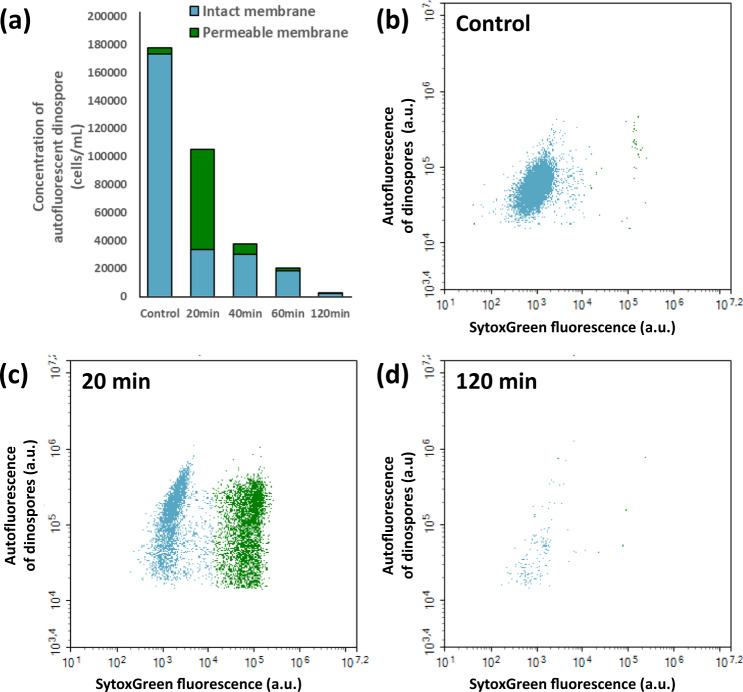
Effects of *A. minutum* filtrate on the density of autofluorescent dinospores and their membrane permeability. Membrane permeability was estimated with the green fluorescence (from 488 nm laser) of cells with SytoxGreen, a stain that only enters cells with damaged permeable membranes. **a** means of the cumulative densities (cells mL^−1^) of autofluorescent dinospores with impermeable (blue) and permeable (green) membranes to the stain. **b** dinospores in the control (stained but not exposed to *A. minutum* filtrate) and exposed to *A. minutum* filtrate for **c** 20 min, **d** 120 min.

In Experiment 3, it was tested whether or not the loss of autofluorescence from dinospores is concomitant to the loss of membrane integrity when exposed to *A. minutum* filtrate. The most potent strain of *A. minutum* (CCMI1002) was used during this experiment. Following the exposure, a rapid decrease in the count of autofluorescent dinospores was observed, with a 40% decrease within 20 min of exposure and a 98% decrease after 2 h (Fig. 5a). This loss of autofluorescent dinospores was preceded by dinospore membrane permeabilization (Fig. 5b–d). After 20 min of exposure to the filtrate, 68% of the still autofluorescent dinospores were permeable to SytoxGreen.

## Discussion

Coculture experiments with *A. minutum* showed that co-occurring resistant dinoflagellates could either decrease survival of the free-living stage of the parasite, or limit infectivity during the second generation, or both. Cells and filtrates of *A. minutum* caused similar effects to the infection dynamic, demonstrating that Dinophyceae can remotely affect parasites through the exudation of APC. Although the lytic activity of the genus *Alexandrium* does not seem related to bacteria [44–46], a role of dinoflagellate microbiome upon excreted APC may exist and should be explored for evidence that bacteria can modulate APC bioactivity. Once released, APC are rapidly diluted, highlighting the importance of cell density and ratios. One may expect a particularly efficient protection for cells in close contact with the APC producers. The formation of dense cell patches with concentrations orders of magnitude higher than background [47–50] is likely more protective at micro-scales as this effect is density-dependent. As effects were observed using filtrates from cultures non-exposed to *Amoebophrya* sp. or its chemical cues, the release of APC appears to be a passive. Despite the passive release of APC, the production of toxins and lytic compounds can induce an extra cost for *Alexandrium* spp. cells under certain conditions [51]. To maximize the fitness of secondary metabolites production, *Alexandrium* cells can modulate lytic potency against microalgae in response to changing physicochemical conditions [52, 53] and toxicity in response to chemical cues from dead microalgal cells [54] or grazers [55]. Accordingly, *Alexandrium* cells are likely to modulate their toxin profile and quantity (including lytic compounds), in the presence of parasites. This hypothesis is further supported by the fact that *A. fundyense* can respond to waterborne cues of *Amoebophrya* sp. by overexpressing genes associated with defensive responses (i.e. production of reactive oxygen species) [56].

*A. minutum* exudates altered the integrity of the membrane prior to the loss of the natural autofluorescence of *Amoebophrya* sp. dinospores. The loss of cell permeability might eventually lead to an osmotic cell lysis. The release of lytic APC by *A. minutum* cells in the phycosphere (i.e., microenvironment surrounding the cells [57]) would act as a protective “shield” and must, at least partially, explain the resistance of *A. minutum* against *Amoebophrya* sp. This strategy was evidently ruled out for some *Amoebophrya* cells, as it has already been reported that the genus *Alexandrium* can be infected by *Amoebophrya* sp. [8, 56]. This could be explained by two hypotheses: (i) either *Amoebophrya* sp. infects only clones of *A. minutum* that do not release APC or, (ii) strategies to counteract APC effects exist in *Amoebophrya*. The second hypothesis has already been proven with *Karlodinium spp*., another potential host [26]. *Amoebophrya* cells can acquire “antidotes” that enable them to avoid toxicity [58]. *Karlodinium* cells produce hydrophobic membrane permeabilizing compounds (Karlotoxins) with bioactivities, and molecular targets that are similar to the permeabilizing compounds from *Alexandrium* [36, 59]. The microalgal cells would be protected from their own toxins by their specific sterol membrane composition [60], a hypothesis also proposed to explain the resistance of *Alexandrium* cells to their own allelochemicals [59]. Cells from *Amoebophrya* sp. do not have a specific sterol signature [27, 61], their sterol composition is rather related to the sterols of the host. The parasite is able to retain host lipid content, including the antidote for toxins, during the infection process. This strategy enables the parasite to avoid cell lysis and to infect the host despite defense mechanisms.

Not all potential hosts are hostiles, however. The APC potency was highly variable between *A. minutum* strains and correlated with anti-microalgal [62] and ichthyotoxic [63, 64] activities. The mode of action of APC is similar to the mode of action of anti-microalgal allelochemicals described from the same strain [36] and from *Alexandrium catenella* (formerly group I of the *A. tamarense/fundyense/catenella* species complex [59]). Both allelochemicals disrupt cell membranes and eventually induce cell lysis. It remains unclear whether APC are the same compounds than the ones described to have anti-microalgal or ichthyotoxic effects or distinct. Their characterization is required to answer this essential question.

Similarly, *S. donghaienis* passively releases APC in the surrounding environment but the potential for active defense remains to be investigated. In comparison with *A. minutum*, a different effect, probably mediated by different molecules, was observed in the presence of *S. donghaienis*. The former species did not affect the survival of the free-living stage of the parasite infecting *S. acuminata*, but rather decreased infectivity (ability to enter the cells) and/or progeny (ability to develop and produce the next generation of dinospores). The production of extracellular bioactive compounds was reported in *S. acuminata* (formerly identified as *S. trochoidea*) [65, 66] but never tested in *S. donghaeinis*. APC may also act indirectly as a signaling system for *S. acuminata* that could, in turn, modify its resistance against *Amoebophrya* sp., a compelling hypothesis that requires more investigation. Importantly, these results emphasize that chemical weapons are not limited to harmful algal bloom species.

It was suggested that the presence of genotypes releasing allelochemicals could facilitate the proliferation of non-allelopathic cells and, therefore, the entire population [44, 67]. Here, it was additionally demonstrated that opportunistic (and competitive) species such as *S. acuminata* could be protected from parasitism and could benefit from a few anti-parasitic producers among *A. minutum* and *S. donghaienis* populations. The cumulative protective effect provided by resistant hosts likely contributes to the survival of a sensitive dinoflagellate species in the presence of parasites, the private good becoming a public good [68]. In cooperative associations, individuals that use common goods produced by others in the absence of feedback are called cheaters. This is the case for non-allelopathic strains of *Prymnesium parvum* that benefit from the exclusion of competitive diatoms by another allelopathic strain [69]. Only the cheaters that are not or weakly sensitive to APC, however, will benefit from the “cure”. For some microalgal species, the APC “cure” might have strong deleterious side effects. At least, a negative effect of *A. minutum* cells (but not of the filtrate) was observed on the growth of *S. acuminata* in cocultures. After all, our results highlight a potential protective role of APC for the dinoflagellate but also suggest that the complexity of planktonic community structure in environmental communities may lead to unexpected outcomes.

APC producers never completely eliminated the parasite, as illustrated by the production of a novel generation of dinospores even in the presence of microalgal cells with a strong APC activity. These results suggest that once inside their host, the parasites may be somewhat protected from APC. Eventually, such chemical defenses that moderate infections could contribute to the maintenance of the parasite in time, whilst avoiding the collapse of host populations. More generally, allelopathy prevents competitive exclusion and promotes biodiversity in phytoplankton by favoring weaker competitors for nutrients [67]. Similarly, APC might promote biodiversity of parasites by favoring the most resistant parasite that may not be the most virulent. Indeed, these results well explain the discrepancies between the virulence of parasites that kill 100% of host cells within few days in the laboratory (this study and others; [23, 70]), and the coexistence of hosts and parasites in ecological studies that include sensitive populations [71, 72]. All of these effects contribute to the explanation of the plankton paradox [73]. Chemical interactions between microorganisms tend to promote biodiversity [67, 74]. They limit the effect of competitive exclusion for nutrients (or hosts for parasites) within the plankton community and could partially explain the coexistence of different parasitic cryptic species competing for the same host as reported by [18].

Despite the ubiquity of the genus *Amoebophrya* sp. in marine ecosystems, many open questions remain about regulation of the parasite dynamic. This study highlighted the release of exudates deleterious to free-life stages of *Amoebophrya* sp. by resistant dinoflagellates. Chemical defenses must play a role in the resistance of dinoflagellates to parasites and more largely a role in their competitiveness. The exudation of anti-parasitic metabolites by resistant hosts in the surrounding environment provides a novel mechanistic link between a host–parasite couple and the surrounding community without the need of physical contact. The exudates not only protect the producer against parasitism but also have the potential to affect the entire community by decreasing propagation of the parasite. This study revealed the importance of the plankton community composition during parasite infection as the severity of the effect fluctuated depending on the species and the strains of the resistant partner, concentrations, and/or the ratios between the different partners. Another factor that has not been assessed in this study but requires further consideration is the potential for chemosensing in these interactions. Some parasites such as the generalist parasite *Parvilucifera sinerae*, can “sense” infochemicals from potential hosts [75], even though they cannot actively select a compatible host [21]. Chemosensing of resistant host infochemicals by a parasite may significantly reduce the efficiency of anti-parasitic defenses and should be studied through micro-scale studies. Although “reductionist” experiments are essential to disentangle interactomes [76], -omic tools will be essential in further studies to identify the APC chemical weapons and assess physiological mode of action. Beside their ecological relevance, the use of APC extracted from dinoflagellates could be a mean to mitigate the parasites with devastating effects on algal mass cultures [77].

## Supplementary information

Figure S1.

Figure S2.

Table S1.

Table S2.

Dataset.
