# Supplementary material for: Dinophyceae can use exudates as weapons against the parasite Amoebophrya sp. (Syndiniales)
Source: ISME Commun. 2021 Jul 12;1:34. doi: 10.1038/s43705-021-00035-x (PMC9723556; doi:10.1038/s43705-021-00035-x)
Supplement: Supplementary file 1 — Figure S1. [file 43705_2021_35_MOESM1_ESM.docx]

**Supporting Information**

Figure S1 : Concentration of fluorescent dinospores over 6 hours after exposure to (a) *A. minutum* CCMI1002, (b) *A. minutum* AM176, (c) *A. minutum* DA1257 and (d-e) *S. donghaienis* Sc39. Dinospores were exposed to ST147 filtrate (control in black), and filtrate at equivalent microalgal densities of 1000 (red lines), 5000 (blue lines) and the maximum concentration of (a, b, c, e) 10000 cells mL^-1^ and (d) 7000 cells mL^-1^ (green lines).
