# Supplementary material for: Dinophyceae can use exudates as weapons against the parasite Amoebophrya sp. (Syndiniales)
Source: ISME Commun. 2021 Jul 12;1:34. doi: 10.1038/s43705-021-00035-x (PMC9723556; doi:10.1038/s43705-021-00035-x)
Supplement: Supplementary file 2 — Figure S2. [file 43705_2021_35_MOESM2_ESM.docx]

**Supporting Information**


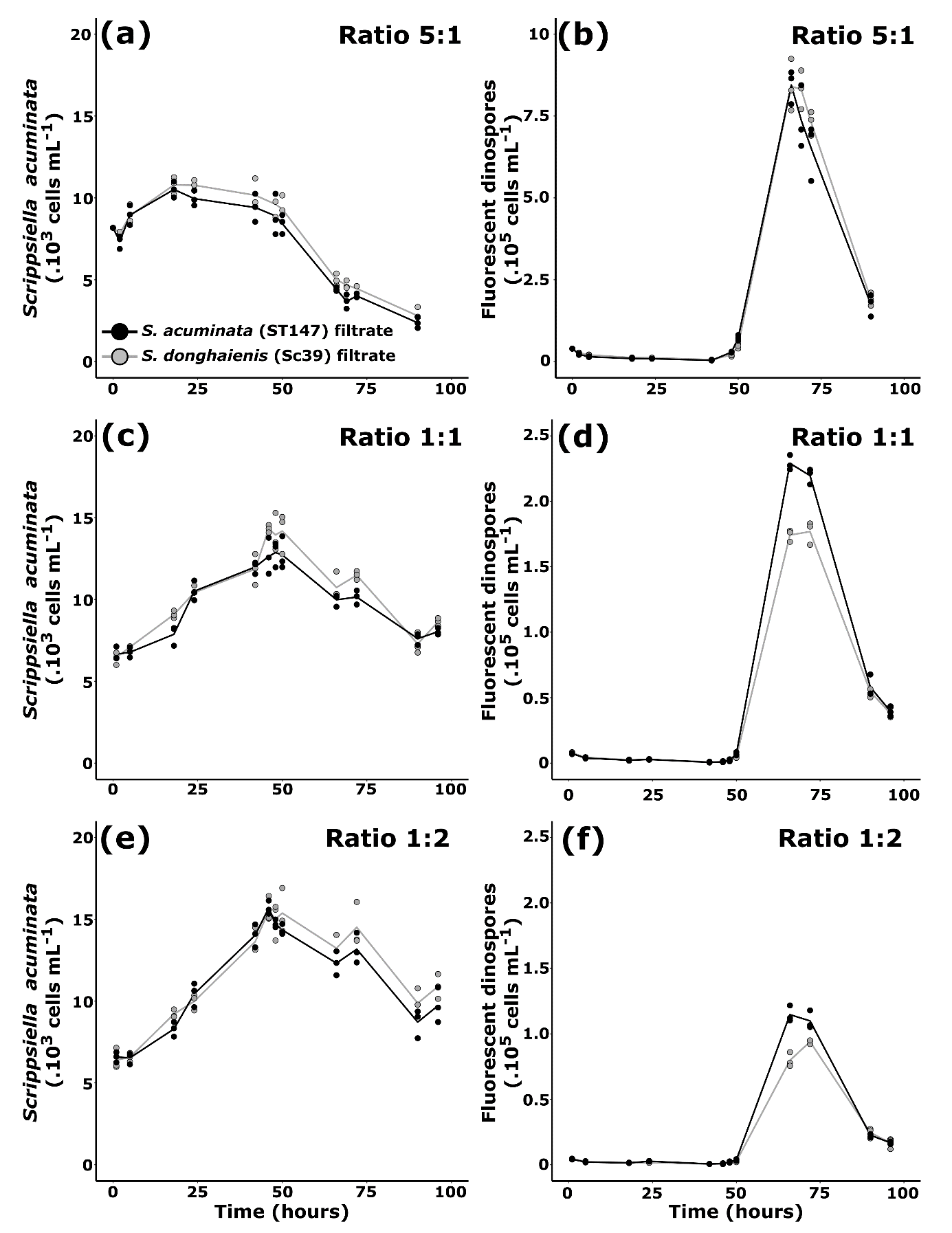


Figure S2: Effect of *Sc39 S. donghaienis* filtrates (Theoretical cell concentration = 7000 cells ml^-1^ for (a) and (b), 10000 cells mL^-1^ for (c, d, e, f) on the infectivity of A25 dinospores to *S. acuminata* (ST147). Infections were performed at three different dinospores: *S. acuminata* ratios; 5:1(a, b),1:1 (C and D) and 1:2 (e, f). The densities of the host ST147 during the infection cycle are shown in graphs (a, c, e). The density of fluorescent dinospores are shown in graphs (b, d, f). The controls (Filtrate ST147) are shown in black while the conditions in presence of Sc39 filtrate are shown in grey.
