# Supplementary material for: Dinophyceae can use exudates as weapons against the parasite Amoebophrya sp. (Syndiniales)
Source: ISME Commun. 2021 Jul 12;1:34. doi: 10.1038/s43705-021-00035-x (PMC9723556; doi:10.1038/s43705-021-00035-x)
Supplement: Supplementary file 3 — Table S1. [file 43705_2021_35_MOESM3_ESM.docx]

**Supporting Information**

Table S1: Details of microalgal and Syndiniales strains used in this study. “/” means that the data is unknown. The numbers indicate the reference (see manuscript) when the information on strain origin or allelochemical potency has already been published.
