# Supplementary material for: Dinophyceae can use exudates as weapons against the parasite Amoebophrya sp. (Syndiniales)
Source: ISME Commun. 2021 Jul 12;1:34. doi: 10.1038/s43705-021-00035-x (PMC9723556; doi:10.1038/s43705-021-00035-x)
Supplement: Supplementary file 4 — Table S2. [file 43705_2021_35_MOESM4_ESM.docx]

**Supporting Information**

Table S2: Effect of culture medium or filtrates on the mortality rate of autofluorescent dinospores of *Amoebophrya* sp. A25 over 24 hours. Loss rate was calculated according to Equation 1 in the manuscript. “NS” means that no significant difference was observed.
